# Practice variation in the Dutch long‐term care and the role of supply‐sensitive care: Is access to the Dutch long‐term care equitable?

**DOI:** 10.1002/hec.3494

**Published:** 2017-03-02

**Authors:** Daisy Duell, Xander Koolman, France Portrait

**Affiliations:** ^1^ Talma Institute, Department of Health Sciences VU University Amsterdam Amsterdam Netherlands

**Keywords:** equity in access, long‐term care, needs assessment, practice variation, supply

## Abstract

Universal access and generous coverage are important goals of the Dutch long‐term care (LTC) system. It is a legal requirement that everyone eligible for LTC should be able to receive it. Institutional care (IC) made up for 90% of Dutch LTC spending. To investigate whether access to IC is as equitable as the Dutch government aspires, we explored practice variation in entitlements to IC across Dutch regions.

We used a unique dataset that included all individual applications for Dutch LTC in January 2010–December 2013 (*N* = 3,373,358). This dataset enabled an accurate identification of the need for care. We examined the local variation in the probability of being granted long‐term IC and in the intensity of the care granted given that individuals have applied for LTC. We also investigated whether the variation observed was related to differences in the local availability of care facilities.

Although our analyses indicated the presence of some practice variation, its magnitude was very small by national and international standards (up to 3%). Only a minor part of the practice variation could be accounted for by local supply differences in care facilities. Overall, we conclude that, unlike many other developed countries, the Dutch system ensured equitable access to long‐term IC.

## 1 INTRODUCTION

In most Western countries, health care expenditure is high and rapidly increasing. Within member countries of the Organisation for Economic Co‐operation and Development (OECD) the United States has the highest health care expenditure (16.9% of the gross domestic product [GDP] in 2015) followed by the Netherlands (10.8% of the GDP in 2015; OECD, 2016). Even though the percentage of individuals older than 65 equals the European average, the Netherlands spends more on long‐term care (LTC) than any other OECD country, namely, 3.7% of its GDP in 2011 (OECD, 2013). To put this percentage into perspective, on average OECD countries devote 1.6% of their GDP to LTC; for instance, Belgium and Germany spend 2% and 1% of their GDP, respectively (OECD, 2013).

Long‐term care is defined as “a range of services needed for persons who are dependent on help with basic activities of daily living” (OECD, 2005). There are three main types of LTC: (a) institutional care (IC; care received in an institution), (b) home care (care received at home or in home‐like settings), and (c) informal care (LTC provided by caregivers from within the client's social network; OECD, 2005). More than 90% of the LTC spending in the Netherlands is currently on IC (Statistics Netherlands, CBS. StatLine, 2016).

The generous LTC system in the Netherlands goes hand in hand with a unique and generous social insurance scheme regulated by the Exceptional Medical Expenses Act (EMEA). 1The EMEA was dissolved on January 1, 2015, and replaced by the Chronic Care Act in order to, amongst other things, reduce the costs of LTC and to diminish geographical differences in entitlements for care (Dutch Court of Audit, in Dutch: de algemene rekenkamer, 2015). The main underlying principle of the scheme is that everyone in need of LTC covered by the EMEA should be able to receive it (Mot E, CPB Netherlands Bureau for Economic Policy Analysis, 2010). In order to achieve uniformity in needs assessment and to guarantee a fair allocation of funds, a nationwide independent committee, the Care Needs Assessment Centre (CNAC), was set up to administer the allocation of LTC covered by the EMEA (Dutch Government, 2005). As the gatekeeper, the CNAC is responsible for assessing requests based on a set of objective criteria set out by the Ministry of Health, Welfare and Sport (CNAC, 2015). Regional care offices are entrusted with ensuring the client that receives the care he or she is entitled to by purchasing and organising services (RIVM. National Public Health Compass, 2013; Mot E, CPB Netherlands Bureau for Economic Policy Analysis, 2010). Regional care offices receive annual budgets for purchasing the entitled LTC (Mot E, CPB Netherlands Bureau for Economic Policy Analysis, 2010). These budgets are based on production agreements made in the previous years, but they are “open‐ended” because care offices receive additional funding if they cannot ensure clients who receive the care they are entitled to within an acceptable time (Dutch Health Authority, in Dutch NZa, 2013). The Dutch Healthcare Authority regulates the market by setting tariffs annually, whereas the Health Care Inspectorate guarantees a minimum level of quality of provided care, as defined by the Dutch government (Mot E, CPB Netherlands Bureau for Economic Policy Analysis, 2010).

Our study assessed whether access to IC in the Netherlands is as equitable as the Dutch government aspires. We focussed on equity in access, defined as individuals with equal needs having equal access to IC irrespective of any ethical judgement or other irrelevant individual characteristics (Goddard & Smith, 2001). More specifically, we assessed the accessibility of IC, given a certain health status or need for care and given that an individual has applied for LTC.

Practice variation (also known as unwarranted variation) is defined as variation in practice styles—usually across regions—that cannot be explained by differences in patient populations such as need or illness, certain risk factors, or patient preferences (Corallo et al., 2014). There is an extensive body of literature devoted to practice variation in different care types and in various institutional contexts. Wennberg (2010) for instance pioneered the study of practice variation in health care and described it in different surgical and diagnostic procedures. Also, there are studies on the variation in health care expenditures between regions, such as the recent one by Chen, Norton, Langa, Le, and Epstein (2014) who investigated the variation in Medicare payments and out‐of‐pocket expenditure between different regions in the United States. There are also a number of studies on practice variation in care for older individuals in various countries and institutional settings (e.g., Kane, Kane, Veazie, & Ladd, 1998; Miller, 2002; Trydegård & Thorslund, 2001). It is remarkable that, to the best of our knowledge, all these studies report large levels of local variation in access and in the use of LTC, even in countries with generous and universal LTC coverage. Some other studies compare the use of LTC between countries. For instance, Bakx, de Meijer, Schut, and van Doorslaer (2015) reported practice variation in the use of informal and formal LTC between Germany and the Netherlands. However, very few studies have investigated practice variation within the Dutch IC setting. One major exception is the recent report of the Dutch Court of Audit (in Dutch: de algemene rekenkamer; 2015). This shows (some degree of) practice variation in the use of institutional and home care across Dutch regions and argues that further research is needed to uncover its determinants in order to reduce regional disparities.

In this study, we primarily focussed on practice variation in needs assessment for Dutch long‐term IC across 32 care office regions. Investigating equity in access across care office regions in the Netherlands is relevant, because LTC budgets are distributed based on these regions (Mot E, CPB Netherlands Bureau for Economic Policy Analysis, 2010). We examined both the probability of being granted an entitlement for long‐term IC and the intensity of the care granted, given that individuals or their agents have applied for LTC. A large number of relevant studies use utilization of care as a proxy for access to care, whereas other factors such as patient or client preferences and local availability of (formal and informal) care may have a large influence on the level of utilization of individuals with granted care (Goddard & Smith, 2001). By analysing care entitlements granted, we were able to more accurately measure equity in access (Goddard & Smith, 2001). It is crucial to control for differences in need in order to characterize practice variation thoroughly and to ensure fair comparisons in access across the Dutch care offices. For this purpose, we used a unique dataset that included all applications for LTC made between January 2010 and July 2014 in the Netherlands and that allowed for a detailed correction for differences in case mix across geographical areas.

There are several potential explanations for practice variation. Chandra, Cutler, and Zirui (2012) mention (a) demand factors (other than need), (b) supply factors, and (c) situational or contextual factors. This paper addresses demand factors, such as a client's socioeconomic status (SES) and determinants such as the price of a certain treatment, but we argue that these factors are less relevant when there is a broad insurance system. Situational or contextual factors relate to matters such as preferred treatment due to a perceived risk of a certain treatment and other behavioural influences based on former experiences. This, however, is difficult to measure and more importantly difficult to influence within the context of policy development. Finally, relevant literature emphasises the importance of supply factors when choosing the appropriate treatment (Sanderson, 1980; Fisher, Wennberg, & Stukel, 2000). Examples of supply factors are (relative) care capacity or available care technologies (Chandra et al., 2012). Therefore, the secondary aim of our study was to investigate whether and to what extent the observed practice variation in long‐term IC is related to differences in the local availability of care facilities. This has yet to be investigated in depth (Dutch Court of Audit, in Dutch: de algemene rekenkamer, 2015). By identifying factors related to practice variation, we might ultimately reduce local inequity in access to Dutch IC.

## 2 DATA AND METHOD

### 2.1 Datasets and study sample

Our dataset contained all CNAC applications and the decisions made in relation to these applications between January 2010 and July 2014 for Dutch LTC covered by the EMEA (*N* = 4,860,007). Most importantly, the dataset provided detailed information on the types and intensity of care granted and a large amount of information on the demographic, health, and social characteristics of applicants. To ensure the privacy of CNAC clients, we removed all information that could be used to identify a specific individual from the dataset and clients were given a unique random identifier. Due to the introduction of a new system for the submission and registration of applications in December 2013, we excluded all applications submitted in January 2014–July 2014. Furthermore, we excluded all applications without a final CNAC decision. Our final dataset included 3,373,358 applications made by 1,415,773 unique clients. 2Clients may have applied several times for identical or different care facilities during the observation window.


The CNAC dataset also provided postal codes for clients' place of residence. This allowed us to retrieve the client's municipality of residence and the care office that processed the client's application. Clients lived in 403 different municipalities, and 32 care offices processed their applications. Information on the municipality of residence enabled us to merge the CNAC dataset with data from ActiZ on the availability of care facilities and providers in each municipality in 2009 (Deuning CM, RIVM, 2009). 3Information on availability of LTC providers per municipality was only available for 2009. Furthermore, to obtain additional information on, for example, average income and the degrees of urbanisation of each municipality, we merged the CNAC dataset with postal code data from statistics Netherlands (CBS; Statistics Netherlands, CBS. Open CBS, 2012). Finally, to obtain data on the intensity of IC granted, we used Dutch Healthcare Authority day tariffs for 2011 for each type of care entitlement. This allowed us to differentiate between the milder and more intensive forms of IC. We provide a detailed description of all relevant variables included in the ActiZ and CBS dataset below.

### 2.2 Dependent variables

Dutch IC is granted using care packages determined according to the severity of the client's needs (in Dutch: ZorgZwaartePakket or ZZP). Our first dependent variable takes the value “1” when the client was granted any form of IC between January 2010 and December 2013 (in other words: any ZZP's) and the value “0” otherwise. Our second dependent variable indicates the intensity of ZZP's granted between January 2010 and December 2013. The intensity of care is measured by means of day tariffs for the IC entitlements granted.

### 2.3 Case‐mix variables

We included the following case‐mix factors into our models: the client's age, gender, reason of application, health and the home circumstances at the time of the application, and the informal and formal care received. All these variables were derived from the CNAC dataset and, except for age, were characterized using dummy variables. In order to avoid excluding too many clients with information missing related to “addictions and other limitations”, we included dummy variables in our models indicating missing information and replaced the missing code by the value 0 in the original dummy variables. All case‐mix variables that were included in our models were variables characterising the health or need of a client.

The models were corrected for age and age squared. Gender took the value “0” for males and the value “1” for females. The following dummies were used to characterize health status at the time of application: (a) physical disability, (b) psychiatric, psychogeriatric, or psychosocial problems, (c) somatic illness, and (d) intellectual disability. The dummy “sensory disabilities” was used as the reference group. The following dummies were used to characterize the reason of application: (a) not specified, (b) new disease, limitation or disorder, (c) change in disease, limitation, or disorder, (d) change in living situation, (e) change in informal care, and (f) end of validity of LTC entitlement. The dummy “other” was used as the reference group. In addition, we included four dummy variables indicating addictions 4To alcohol, soft drugs, and hard drugs. and/or other limitations, which took the value “1” in case of (a) no addiction or limitations, (b) mild addictions or limitations, (c) moderate addictions or limitations, and (d) serious addictions or limitations and the value “0” otherwise. Four dummy variables were used to characterize the informal care a client received at the time of the application for LTC and took the value “1” when a client (a) had almost no help, (b) had help one or more times per week, (c) had help daily or almost daily, or (d) when unknown and the value “0” in case of no help. Other forms of granted LTC were also included in our model by means of five dummy variables taking the value “1” in case of being granted (a) nursing home care, (b) personal care, (c) care including stay, (d) care including a treatment, and (e) counselling and the value “0” otherwise. The home circumstances at the time of application were characterized using the following seven dummy variables, which took the value “1” when a client lived in (a) a multiperson household, (b) a household with a partner and children, (c) independently but with a partner, (d) a household consisting of an adult with one or more children, (e) alone and independently, (f) as a child living in the parental home with his or her parents, or (g) where the situation was unknown. The category “clients living in an institution” was used as the reference group. Interaction variables were also included in the models. These were interactions between, for example, age and gender, age and dominant health problems, and gender and dominant health problems that could also affect the decision made by the assessors. Finally, only for the analyses on the intensity of IC granted, dummy variables characterizing the type of entitlements granted were included in the model.

### 2.4 Availability and characteristics of LTC providers per municipality

Information on the availability of LTC providers derived from the ActiZ data included the number of institutions providing part‐time care, the number of institutions providing homecare, the number of beds per nursing home, and the percentage of persons employed in the health care sector per municipality. We corrected the number of facilities for the number of inhabitants older than 65 per municipality to make these variables comparable across municipalities. Furthermore, this dataset also provided information on the number of hospitals within 20 km of the municipality of the client's residence and the number of general practitioner (GP) practices within 5 km. Note that we did not have data on the exact location of the care facilities within each municipality. Therefore, we were not able to directly calculate the distance between a client's home and the care facilities. Instead, we used the degree of urbanisation of the municipality of residence, derived from the dataset of Statistics Netherlands, as a proxy for the average distance between the client's home and his or her health care facilities. We classified a municipality as urban when it included more than 1,000 addresses per square kilometer.

### 2.5 Statistical analyses

#### 2.5.1 Descriptive statistics

We computed descriptive statistics of the CNAC clients and of the supply of care facilities. To check for differences in population characteristics across care office regions, we used analysis of variance. Thereafter, in order to explore the practice variation across care offices, we performed several regression analyses.

#### 2.5.2 Assessing practice variation

In the first step of our analyses, we used logit and truncated regression models to analyse whether differences in case‐mix could explain differences across care office regions in receiving IC entitlements (given that an individual had applied for it). We assumed that any variation in outcomes that was explained by the case‐mix variables was “warranted” (i.e., not inequitable) and that any variation in outcomes that was not explained by the case‐mix variables was “unwarranted” and therefore inequitable. We used regression models with a correction of the standard errors for clustering of individuals in order to control for within‐cluster correlation that may be present because individuals can apply more than once for IC. Logit regression was used for the binary dependent variable indicating any form of IC granted, whereas truncated regression models were used for the continuous outcome variable “day tariffs for IC entitlements granted.” The case‐mix variables are described in subsection 2.3.

In order to make sure that the regional characteristics did not bias the coefficients of the case‐mix variables, we decided not to include care office region fixed effects in the first step of the analyses. We believe that the regional characteristics otherwise could act as a mediator between the case‐mix variables and the outcome variable. The resulting case‐mix effects could then be viewed as partial (or conditional) effects and would not capture the full effect of the case‐mix. Furthermore, we included in all models month dummies indicating the timing of the application to account for possible changes in contextual conditions over time. All regression analyses can be summarized by means of the following Equations (1) and (2):

(1) $$\text{prob}\;\left(y_{j}^{1}=1\right)=\frac{1}{1+e^{-\left(\beta_{0}^{1}+\sum_{i=1}^{I}X_{\mathrm{ij}}\beta_{i}^{1}+\sum_{k=1}^{K}M_{\mathrm{kj}}\gamma_{k}^{1}\right)}},$$

(2) $$y_{j}^{2*}=\beta_{0}^{2}+\sum_{i=1}^{I}X_{\mathrm{ij}}\beta_{i}^{2}+\sum_{k=1}^{K}M_{\mathrm{kj}}\gamma_{k}^{2}+u_{j}^{2},$$

$$y_{j}^{2}=y_{j}^{2*}|\;y_{j}^{2*}>0,$$

where

$y_{j}^{1}\;$: = Dummy variable indicating whether client *j* received any IC entitlement
$y_{j}^{2}$: = Day tariff per IC entitlement granted to client *j*
*X*_*ij*_: = Case‐mix variable *i* for client *j*
*M*_*kj*_: = Dummy for month of application k for client *j*
*I*: = Total number of case‐mix variables *i*
*K*: = Total number of months *k* in the observation window
$\beta_{0}^{1}$, $\beta_{i}^{1}$, $\gamma_{k}^{1}$: = Parameters for the probability of being granted an IC entitlement
$\beta_{0}^{2}$, $\beta_{i}^{2}$, $\gamma_{k}^{2}$: = Parameters for the day tariff of the IC entitlement granted
$u_{j}^{2}$: = Stochastic error term for the day tariff per IC entitlement for client *j*.

In the second step of the analyses, we calculated, using the estimation results of the first stage, the predicted probabilities and the intensity of care that each individual would be expected to have based on his or her care need (as captured by the right‐hand side variables in the first stage). In order to determine the magnitude of practice variation between care office regions, we computed the mean difference between the observed values and the predicted values for each care office region and their corresponding confidence intervals. To make it more easily interpretable, we added the average value for the whole population to all mean differences (see Equation (3)).

(3) $${\mathrm{PV}}_{r}^{1,2}=\sum_{r}\left(y^{1,2}-\hat{y^{1,2}}\right)/N_{r}+\hat{y^{1,2}}.$$

Using the same notations as above (after exclusion of the index *j*) and where:

${\mathrm{PV}}_{r}^{1,2}$: = Practice variation (*PV*) as the case‐mix adjusted average of (a) probability of acceptance and (b) day tariff for region *r*
*y*^1 , 2^: = Observed value
$\hat{y^{1,2}}$: = Predicted values based on case mix
*N*_*r*_: = Number of records in region *r*

In order to improve our prediction, we corrected the standard errors using a parametric bootstrap procedure based on 100 replications. The bootstrap method was used in order to assess sampling variability while allowing for the use of estimated measurements by incorporating these estimations in the bootstrap procedure (van Doorslaer, Koolman, & Jones, 2004). If we calculate the practice variation measurements without bootstrapping, it might lead to measurements with downward biased standard errors, to an underestimation of the confidence intervals and thus to an overestimation of the practice variation.

If the difference between observed and predicted values was significantly different from the population average, then part of the variation in IC entitlements could not be explained by differences in client population across care office regions. There was statistical evidence of practice variation when the confidence intervals of the care office regions, based on the statistical significance level of 0.05, did not overlap.

#### 2.5.3 Explaining practice variation by local availability of care facilities

Finally, in the third step of the analyses, we analysed whether the availability of care was related to the observed practice variation in the Dutch IC. Because we had access to information on availability of LTC at the level of the municipality, we estimated a linear regression model with the mean difference between the observed and predicted values per municipality as a dependent variable and the variables characterizing the availability of LTC as independent variables using a statistical significance level of 0.05 (see subsection 2.4). These analyses can be summarized by means of Equation (4):

(4) $${\mathrm{PV}}_{m}^{1,2}=\beta_{0}^{1,2}+\sum_{l=1}^{L}S_{\mathrm{lm}}\beta_{l}^{1,2}+u_{m}^{1,2}.$$

Using the same notation as before and where

${\mathrm{PV}}_{m}^{1,2}$: = Practice variation for municipality *m*
*S*_*lm*_: = Supply of care facilities variable *l* for municipality *m*
*L*: = Total number of supply of care facilities variables *l*
$\beta_{0}^{1,2},$$\beta_{i}^{1,2}$: = Parameters
$u_{m}^{1,2}$: = Stochastic error term for municipality *m*

## 3 RESULTS

### 3.1 Descriptive statistics

The dataset includes 3,373,358 applications administered by 32 care offices in 403 municipalities. A ZZP/entitlement for IC was granted in 29% of the applications. The day tariff for an IC entitlement granted was on average €150.10. Table 1 provides more detailed information on the number of applications and outcome variables.

**Table 1:** Outcome variables

Variable name	2010	2011	2012	2013	Total
Percentage of observations for which an IC entitlement (ZZP) has been granted	30%	30%	30%	25%	29%
Mean NZa day tariff IC (ZZP)	€151.35	€153.19	€143.30 (7%)^a^	€151.33 (1%)^a^	€150.10 (2%)^a^
Number of applications	877,088	856,115	856,328	783,827	3,373,358

*Note*. IC = Institutional care; NZa = Dutch Health Authority; ZZP = ZorgZwaartePakket.

a Percentage of missing observations shown between brackets.

**Graph 1:**
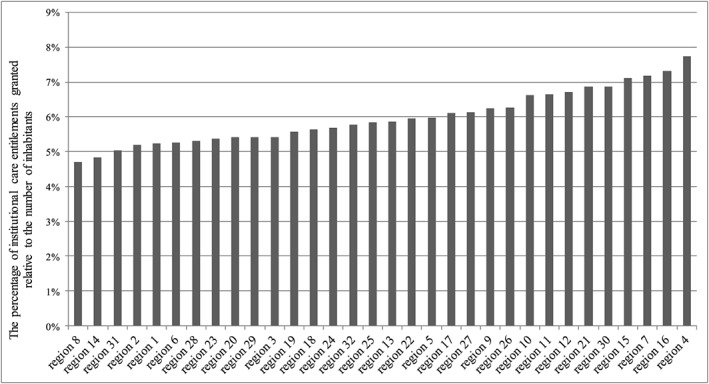
Percentages of institutional care entitlements granted relative to the number of inhabitants per care office region

Graph 1 illustrates the distribution of ZZP's entitlements among care office regions relative to the number of inhabitants. It indicates differences in the percentage of IC entitlements across care office regions (lowest region: 4.7%; highest region: 7.7%).

Table 2 provides information on client characteristics. Institutional care entitlements were granted to 1,415,773 unique clients. These clients were, on average, 66 years old, and the majority of the clients were female. Most clients' dominant health problem was a somatic illness. The *p* values derived from one‐way analysis of variance analyses confirmed large differences in case mix across care office regions.

**Table 2:** Client characteristics

Variables	Total
Number of unique clients: 1,415,773	
Mean age clients	65.9
Gender: % Males	41.7
Dominant health problem (in %)	
Physical disability	3.9
Psychiatric, psychogeriatric, or psychosocial problems	17.2
Somatic illness	69.1
Intellectual disability	8.5
Sensory disabilities	0.9
Addictions and/or limitations (in %)	
No addiction	30.8^b^
No limitation	27.6^b^
A serious addiction	1.0^b^
A serious limitation	5.1^b^
Informal care (in %)	
Daily or almost daily	14.0^c^
No help	12.6^c^
Receiving other forms of LTC besides an IC entitlement (in %)	
Personal care	3
Nursing home care	1
Counselling	1
Reason of application (in %)	
New disease, limitation, or disorder	21.7
Change in disease, limitation, ordisorder	17.0
End of validity of LTC entitlement	13.1
Living situation (in %)	
Lives independently but with partner	26.5 (1)^a^
Lives alone and independently	47.4 (1)^a^
Lives in an institution	12.6 (1)^a^
Proxy for SES (average income per district)	
Mean income per district	€13,200 (1)^a^
Average score for self‐reliance^c^	
Social skills	20.1 (56)^a^
Moving	21.1 (54)^a^
Personal care	8.5 (53)^a^
Domestic life	20.8 (74)^a^
Social relationships and social life	10.3 (68)^a^
Orientation disorders	2.9 (61)^a^
Problem behaviour safety	3.7 (61)^a^
Psychosocial functioning	3.9 (58)^a^
Psychosocial well‐being	7.2 (62)^a^

*Note*. LTC = long‐term care; IC = institutional care. Results from a one‐way ANOVA test across care offices show a p‐value smaller than 0.0000 for all variables.

a Percentage of missing observations shown between brackets.

b A dummy variable was included indicating the missing values.

c Average scores on self‐reliance on a scale from 0 to 100: 0 = *fully independent* and 100 = *fully dependent* (CNAC & HHM, 2008).

Table 3 shows the characteristics of supply of care facilities per municipality. Most importantly, 57% of the clients lived in an urban district with more than 1,000 addresses per square kilometer. Furthermore, nursing homes had, on average, a capacity of 13.6 beds per 1,000 inhabitants older than 65.

**Table 3:** Supply characteristics per municipality

Variables	Total
Number of unique municipalities: 403	
Degree of urbanisation (percentage of ≥1,000 addresses per km^2^)	57
Mean number of institutions with part‐time care per 1,000 inhabitants older than 65	0.75
Mean number of institutions with homecare per 1,000 inhabitants older than 65	0.19
Mean number of beds or nursing home per 1,000 inhabitants older than 65	13.6
Percentage of persons employed in the health care sector or total persons employed in a municipality	14
Mean number of hospitals within 20 km of the municipality where the client lives	5
Mean number of GP practices within 5 km of the municipality where the client lives	20

*Note*. GP = general practitioner.

### 3.2 Results of practice variation across care office regions

**Graph 2:**
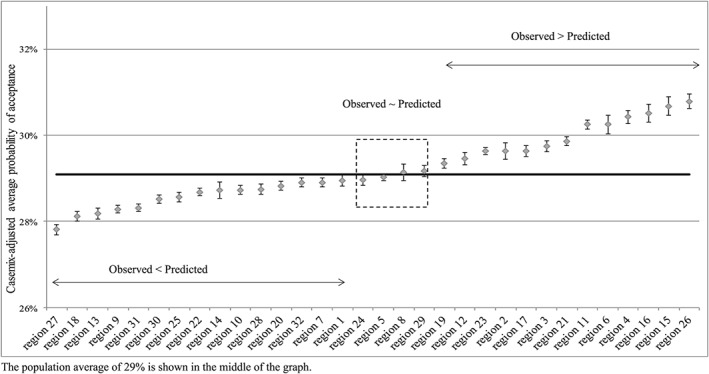
Practice variation in institutional care entitlements granted

Graph 2 shows the difference in the predicted and the observed probability of receiving a ZZP/entitlement for IC per care office region. This illustrates the magnitude of the practice variation, in other words, the equity in access to Dutch IC. The pseudo R‐squared of the model is 0.6775. The results indicate that the predicted and the observed probabilities were significantly different from the population average in all regions except for regions 24, 5, 8, and 29. The regions, in which the observed and predicted probabilities were not significantly different from the population average, are ring fenced in the middle of the graph. Furthermore, our results show practice variation between many regions, as most confidence intervals do not overlap. The magnitude of practice variation was about 3% at a population average of 29% (the variation ranges from −1.28% to +1.70%).

**Graph 3:**
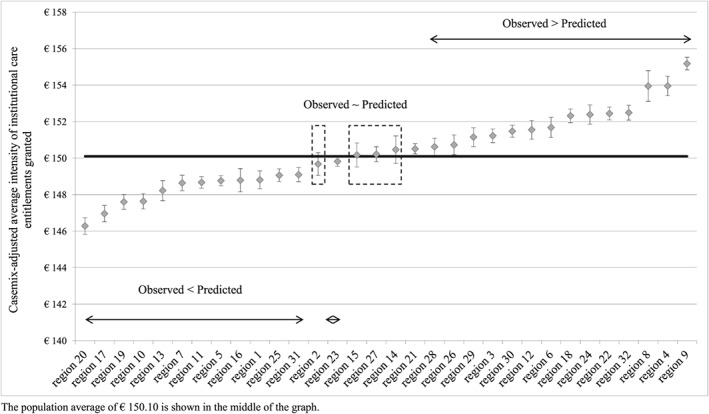
Practice variation in the intensity of IC granted

Graph 3 shows the difference in the predicted and the observed day tariffs for IC entitlements, given that an IC entitlement has been granted. The estimated R‐squared of this model is 0.3864. The predicted and the observed values were not significantly different from the population average for care office regions 2, 15, 27, and 14. These regions are ring fenced in the middle of the graph. In all other regions, our estimates deviate from the observed day tariffs for IC. Again, our results showed practice variation between regions, as confidence intervals between regions did not always overlap. In day tariffs, the magnitude of practice variation between regions was about €8.90 (range of €−3.82 to €+5.08), with a population average of €150.10. Note that the care office regions are ranked differently in 2 and 3.

Table 4 reports the results of the third step of our analyses on the association between supply factors and practice variation. This table shows that the variables “number of institutions with part‐time care” and “number of hospitals within 20 km” were positively associated with the practice variation in the probability of receiving an IC entitlement. Furthermore, it shows that “the number of institutions with part‐time care” was negatively associated with the practice variation in day tariffs of IC and that “the percentage of persons employed in the health care sector” was positively associated with the practice variation in day tariffs of IC granted.

**Table 4:** Estimation results practice variation and local availability of care

Variables	${\text{Coefficients}\;\mathrm{PV}}_{m}^{1}$	${\text{Coefficients}\;\mathrm{PV}}_{m}^{2}$
Degree of urbanisation (percentage of ≥1,000 addresses per km^2^)	0.0019	−0.2664
Number of institutions with part‐time care per 1,000 older than 65	0.0044***	−2.0435***
Number of institutions with homecare per 1,000 older than 65	−0.0009	0.1327
Number of beds or nursing home per 1,000 older than 65	0.0000	−0.0088
Percentage of persons employed in the health care sector/total persons employed in a municipality	0.0003	0.2325**
Number of hospitals within 20 km	0.0012***	−0.0214
Number of GP practices within 5 km	0.0000	0.0043

*Note*. GP = general practitioner; IC, institutional care.

${\mathrm{PV}}_{m}^{1,2}$ = Practice variation for municipality *m*, for 1: any IC entitlement granted or 2: day tariff IC entitlement granted.

* *P* < .1.

** *P* < .05.

*** *P* < .01.

### 3.3 Sensitivity analyses

We performed several sensitivity analyses. First, we repeated the analyses after only including applications performed by care providers 5In some cases, health care suppliers are mandated to administer the application on behalf of the CNAC (CNAC, 2014). (*N* = 1,495,439). This was to investigate whether the practice variation increased when only applications that were not checked by the CNAC were included in the analyses. Second, we investigated the association between a client's SES and practice variation in IC entitlements. Even though supply factors were our main focus, clients' SES could also affect the process of allocation of LTC and, in turn, explain part of the observed practice variation. Due to its administrative nature, our database did not include variables related to SES. Therefore, we were forced to use a proxy “average income per district” 6A municipality contains multiple districts. provided by Statistic Netherlands. Third, we included extra information on the clients' health (namely, average scores for self‐reliance). These variables were not included in our main analyses because of their high percentage of missing cases (around 80%). Fourth, we computed the mean difference between the observed and the predicted entitlements in absolute values per care office region. That was to investigate whether our conclusions on practice variation were affected by not taking into account that the differences between the observed and predicted values were sometimes positive, sometimes negative. Both indicate some level of practice variation (namely, too many or too few entitlements), but, by averaging these differences by region, we may underestimate the actual level of practice variation between regions. We were therefore expecting that, when using absolute values, it would lead to an increase of the overall practice variation per region. This analysis gives additional insight into the level of variation within care office regions.

Table 5 shows the main results derived from the sensitivity analyses. After (a) only including applications by health care providers into our model and (b) including income as a proxy of SES, the results show great similarity with the ones of 2. Although the order of the regions was somewhat different as compared to 2, the size of the practice variation remained around 3%. In addition, in most of the regions, the predicted and observed probabilities were statistically significant, different from the population average. This confirmed that practice variation was present and that its magnitude was similar to that in our previous calculations. The results from our third sensitivity analysis, where variables on self‐reliance were added and consequently a large number of observations (around 80%), were excluded, showed larger confidence intervals and a few more of them overlapped compared to our main analyses. When comparing the results of our analysis including the self‐reliance scores and our main analysis, we observed a different pattern for the regions. Although this analysis also verified the presence of practice variation, its magnitude was slightly smaller, namely, 2%. The results from our fourth sensitivity analysis indicate that the variation within the regions, between the observed and predicted model, was somewhat larger when compared to our initial results. However, the variation between the regions was still relatively small.

**Table 5:**
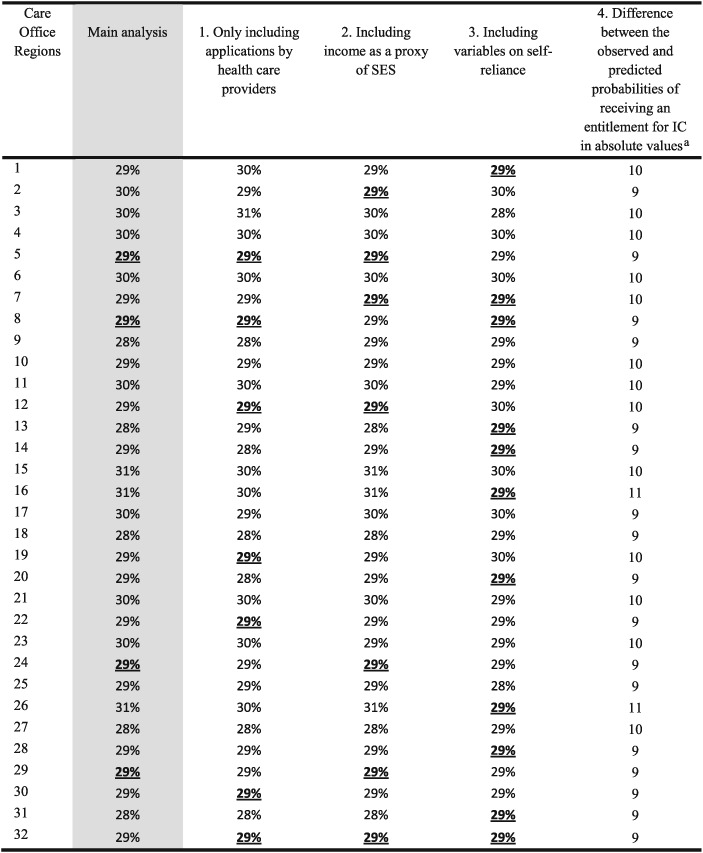
Sensitivity analyses: practice variation in IC granted (in %)

*Note*. IC = Institutional care; SES, socioeconomic status. The underlined bold percentages show in which regions we find statistical evidence of practice variation.

The absolute values are presented as the deviation from zero.

## 4 DISCUSSION AND CONCLUSION

The primary aim of our paper was to investigate whether the principle of equitable access to long‐term IC is achieved in the Netherlands. After thoroughly correcting for differences in need across geographical areas and quantifying the level of practice variation for those who had applied for LTC, we conclude that the probability of receiving long‐term IC and the associated intensity of care granted depend on the region where the client lives. However, the magnitude of the variation is very small compared to the results from other studies. Although we found that the probability of being granted IC differed on average by 3% between Dutch regional care offices, Trydegård and Thorslund (2001) found that in Sweden, for people aged 80 years and older, coverage for home help differed by 47% and coverage for special housing differed by 28% across municipalities, even though Sweden also strives towards equitable access to IC. When looking at non‐IC, García‐Gómez, Hernández‐Quevedo, Jiménez‐Rubio, and Oliva‐Moreno (2015) found that in Spain, the distribution of LTC use and unmet need was not equitable. Furthermore, high degrees of practice variation in LTC were reported, for example, in the United States (up to a difference of 12.3% in the older individuals receiving care from the Medicaid program [Miller, 2002]). Clearly, the fact that different degrees of practice variation in LTC and evidence for unequitable LTC were found across countries could be due to differences in methodology or case‐mix corrections used in the studies. The fact that we do not find large differences in access to Dutch IC across regions is in line with the findings of Bakx et al. (2015) and de Meijer, Koopmanschap, Koolman, and van Doorslaer (2009). Bakx et al. (2015) show that access to Dutch LTC is independent of one's income, but in Germany, the opposite was found. De Meijer et al. (2009) also show that having lower income in the Netherlands does not restrict access to LTC. Our findings are also in line with the descriptive study by Mot, Faber, Geerts, and Willemé (2012), which concludes that, based on all its characteristics, the Dutch LTC system scores higher in terms of equity in access than other European countries.

Practice variation in the Dutch curative system seems to be much larger than in the Dutch LTC system. For instance, the numbers of hip and knee replacements due to arthritis differ between hospitals by a factor 2.2 and 3.3, respectively (Vektis & Plexus, 2011). The methods used by Vektis and Plexus (2011) and our study are comparable; therefore, the disparity is likely due to differences in the way these health services are organized and financed. Although LTC is regulated centrally through a social insurance system in the Netherlands, curative care is organised through a private insurance scheme. And although the Dutch LTC system is primarily managed by the government, the curative system is managed by health insurers through a system of regulated competition. We therefore would expect less variation in the LTC sector as compared to the curative sector where variation is likely to arise between clients of different health insurers.

We used a large national population‐wide database for this research, which enabled us to thoroughly correct for differences in client population across geographical areas and to perform a detailed research on practice variation in the Dutch LTC. This is a major strength of our paper. Even though we had the rich and detailed CNAC dataset at our disposal, we cannot conclusively reject the possibility that some dimensions of the case mix are still unobserved and not included in our models. If this is the case, we suspect that the true degree of practice variation in access to Dutch IC would be even smaller than the one found in the current paper. In addition, we performed several sensitivity analyses in order to investigate how some other case‐mix factors (self‐reliance scores and socioeconomic factors) influenced the results of our main analysis. All sensitivity analyses showed similar results, indicating that these factors do not significantly affect our main conclusions.

Given the many case‐mix variables in our regression models, we acknowledge that we might unintentionally correct for differences caused by undesirable factors. For instance, the sex of a client. This variable was included because of the biological differences in health between men and women, which in turn could lead to different diseases and different types of LTC needs. In addition, the sex of a client may reflect cultural differences influencing whether one is able to live at home or should be institutionalized. Older women may have been at home for a large part of their lives taking care of their husbands. We therefore believe men and women to be at different health stages when the decision is made if one is able to live at home or should be institutionalized. Aside from the above reasoning, one could also argue that by including the sex of a client, we unintentionally correct for sexist attitudes favouring one group over the other resulting in inequitable differences. However, this factor is included in many studies on practice variation, for example, in studies performed by the leading institute on practice variation “The Dartmouth Institute for Health Policy and Clinical Practice” (Skinner & Fisher, 2010). In addition, in order to achieve uniformity in needs assessment, the CNAC is responsible for assessing requests based on a fixed set of objective criteria set out by the Ministry of Health, Welfare and Sport (CNAC, 2015). The need assessors are trained nurses with additional training to follow these rules objectively. Their performance is monitored, and working procedures involve several feedback loops to minimize undesirable variation or any kind of discrimination (such as cultural, ethnic, or religious). Therefore, we made the assumption that the professionals who process LTC applications are able to assess need adequately.

Furthermore, because we do not have information on individuals who may have been eligible for IC but did not apply, we may underestimate the true level of practice variation. However, the CNAC dataset shows that only about 5% of the applications for IC in the Netherlands were initiated in 2013–2014 by the client. Sixty‐five percent of the applications were initiated by the GP or (outpatient) LTC providers. In the Netherlands, the GP was likely to actively treat all eligible people and functioned as a “gatekeeper,” referring patients to secondary care. Moreover, visiting the GP and/or applying for care at CNAC were free of charge. Also, income‐related inequity plays little to no role in the probability of a GP visits. Consequently, virtually all people in need of IC are undergoing treatment in the Dutch health care system. Finally, individuals who have been granted care may opt to postpone its use or decide not to use it at all. All this is likely to decrease the likelihood of nonsubmission for IC. However, even if the number of eligible people who did not apply for care is likely to be low in the Netherlands, regions with higher percentages of, for example, socially deprived individuals may face higher percentage of nonsubmission than others. This is a very interesting topic for future research.

All of the above allows us to conclude that the small variation we found compared to other countries might be thanks to the design of the Dutch LTC system.

Our findings also suggest that factors other than need, such as supply factors, could explain some of the variation in access to the Dutch IC. As expected, mainly positive associations are found, indicating that more supply was associated with more practice variation. But interestingly, we found a positive association between the number of institutions with part‐time care and the probability of being granted an entitlement and a negative association between the same variable and the intensity of the granted IC. This could be explained by the following: Some clients are granted access to IC but can for the time being manage without it. If part‐time care can prevent institutionalisation, this would lead to more variation in the probability of receiving an entitlement. The magnitude of our coefficients on supply is relatively small compared to those described in other relevant literature. For instance, Fisher et al. (2000) showed that for regions in the United States, a higher number of hospital beds were associated with a higher rate of hospitalisation: An individual is on average up to 30% more likely to be hospitalized when living in a region with a lot of hospital beds (4.5 hospital beds per 1,000 residents; Fisher et al., 2000). The fact that the magnitude of the coefficients from our results on supply was relatively small is in line with our expectations because we believe that, if supply factors played a large role in Dutch IC, we would have observed more practice variation. Furthermore, because we did not have information on individuals who needed IC but did not apply for LTC, we have to keep in mind that there may also be a relation between the probability of applying for LTC and supply.

Our results indicate that (a) practice variation is relatively small in magnitude and that (b) supply factors are not strongly related to the variation and lead us to conclude that the principle of equity in access to the Dutch IC is carried through in practice. Therefore, the organizational framework of the Dutch social insurance scheme could be used as a model for other health care systems for LTC that strive for equity in access. However, this unique system comes with high costs. These high costs in combination with an ageing population create difficult challenges of, on the one hand, the need for containing health care costs, and, on the other hand, the goal of providing equitable access to high quality IC.

## ACKNOWLEDGEMENT AND FINANCIAL SUPPORT

We thank the Dutch Care Needs Assessment Centre (CNAC) for providing us with access to the data and for facilitating the study. Although this study has been partly financed by the CNAC (because one of the researchers has a 1‐day a week appointment as a researcher with the CNAC), we would like to emphasize that this study does not necessarily reflect the view of CNAC on unexplained practice variation.

## ETHICS APPROVAL

According to the Dutch law, a study like this does not require approval from an Ethics Review Board, as the study does not fall within the scope of the Medical Research Involving Human Subjects Act (WMO).

## Supporting information

Data S1. Supporting info item
