# Supplementary material for: Practice variation in the Dutch long‐term care and the role of supply‐sensitive care: Is access to the Dutch long‐term care equitable?
Source: Health Econ. 2017 Mar 2;26(12):1728–42. doi: 10.1002/hec.3494 (PMC5811918; doi:10.1002/hec.3494)
Supplement: Supplementary file 1 — Data S1. Supporting info item [file HEC-26-1728-s001.zip › Additional Web Appendix.doc]

**Additional Web Appendix**

Even though regression outputs in the first step of the main analysis cannot be directly used to draw conclusions on practice variation between regions we have uploaded the output from our main Logit regression to give more insight into the Results.

**Additional table.** Logistic regression analyses (output before bootstrapping)

| **Y= Institutional LTC Entitlement** | **Coefficient** | **Robust Standard Error** | **z** | **P> z** | **95% Confidence**  **Interval** | |
| --- | --- | --- | --- | --- | --- | --- |
| Informal care almost no help | -.1244743 | .0142529 | -8.73 | 0.000 | -.1524094 | -.0965392 |
| Informal care one or more | -.1531557 | .013821 | -11.08 | 0.000 | -.1802443 | -.1260672 |
| Informal care daily | -.5587662 | .0100365 | -55.67 | 0.000 | -.5784375 | -.539095 |
| Informal care unknown | .1892524 | .0070302 | 26.92 | 0.000 | .1754736 | .2030313 |
| Other addiction no | -.0530211 | .0189286 | -2.80 | 0.005 | -.0901205 | -.0159218 |
| Other addiction mild | .0894854 | .0239362 | 3.74 | 0.000 | .0425712 | .1363996 |
| Other addiction moderate | .2876333 | .0255776 | 11.25 | 0.000 | .2375022 | .3377644 |
| Other addiction serious | .4577362 | .0343285 | 13.33 | 0.000 | .3904536 | .5250188 |
| Other addiction not measured | -.4034722 | .2334305 | -1.73 | 0.084 | -.8609876 | .0540433 |
| Use of alcohol no | -.0006876 | .023128 | -0.03 | 0.976 | -.0460175 | .0446424 |
| Use of alcohol mild | .0667579 | .0224212 | 2.98 | 0.003 | .0228132 | .1107026 |
| Use of alcohol moderate | -.0342518 | .0224564 | -1.53 | 0.127 | -.0782655 | .0097619 |
| Use of alcohol serious | .0968156 | .0283578 | 3.41 | 0.001 | .0412354 | .1523958 |
| Use of alcohol not measured | -.27747 | .2542638 | -1.09 | 0.275 | -.7758178 | .2208779 |
| Use of hard drugs no | .075779 | .0290312 | 2.61 | 0.009 | .018879 | .132679 |
| Use of hard drugs mild | -.0365587 | .0303307 | -1.21 | 0.228 | -.0960059 | .0228885 |
| Use of hard drugs moderate | -.0454364 | .0324315 | -1.40 | 0.161 | -.1090009 | .0181282 |
| Use of hard drugs serious | -.0707078 | .037866 | -1.87 | 0.062 | -.1449237 | .0035082 |
| Use of hard drugs not measured | .7745536 | .3603371 | 2.15 | 0.032 | .0683059 | 1.480.801 |
| Use of soft drugs no | -.0187795 | .0258436 | -0.73 | 0.467 | -.0694321 | .0318731 |
| Use of soft drugs mild | .2536073 | .0249052 | 10.18 | 0.000 | .204794 | .3024206 |
| Use of soft drugs moderate | .3907441 | .0255474 | 15.29 | 0.000 | .3406722 | .4408161 |
| Use of soft drugs serious | .6231609 | .0316201 | 19.71 | 0.000 | .5611866 | .6851353 |
| Use of soft drugs not measured | -.3088753 | .2738248 | -1.13 | 0.259 | -.8455619 | .2278114 |
| Limitation head balance no | .2032441 | .0207295 | 9.80 | 0.000 | .1626151 | .2438731 |
| Limitation head balance mild | .088214 | .0297559 | 2.96 | 0.003 | .0298936 | .1465344 |
| Limitation head balance moderate | -.0220026 | .0496436 | -0.44 | 0.658 | -.1193022 | .075297 |
| Limitation head balance serious | -.3669316 | .1044887 | -3.51 | 0.000 | -.5717256 | -.1621376 |
| Limitation head balance not measured | rd-.6247935 | .3095355 | -2.02 | 0.044 | -1.231.472 | -.0181149 |
| Limitation torso balance no | .1416602 | .0214117 | 6.62 | 0.000 | .099694 | .1836265 |
| Limitation torso balance mild | .305644 | .0175541 | 17.41 | 0.000 | .2712386 | .3400493 |
| Limitation torso balance moderate | .6657527 | .0204174 | 32.61 | 0.000 | .6257352 | .7057701 |
| Limitation torso balance serious | .8031688 | .0511816 | 15.69 | 0.000 | .7028547 | .9034829 |
| Limitation torso balance not measured | urd.6050338 | .268898 | 2.25 | 0.024 | .0780034 | 1.132.064 |
| Limitation arms no | -.147418 | .0285205 | -5.17 | 0.000 | -.2033172 | -.0915188 |
| Limitation arms mild | -.0778303 | .0266568 | -2.92 | 0.004 | -.1300766 | -.0255841 |
| Limitation arms moderate | .0741217 | .0294098 | 2.52 | 0.012 | .0164796 | .1317639 |
| Limitation arms serious | .4444428 | .0625447 | 7.11 | 0.000 | .3218575 | .5670282 |
| Limitation arms not measured | -.1456176 | .5654025 | -0.26 | 0.797 | -1.253.786 | .9625509 |
| Limitation legs no | -.2788182 | .031291 | -8.91 | 0.000 | -.3401474 | -.217489 |
| Limitation legs mild | -.1353464 | .0248188 | -5.45 | 0.000 | -.1839903 | -.0867024 |
| Limitation legs moderate | .3337977 | .026333 | 12.68 | 0.000 | .2821859 | .3854095 |
| Limitation legs serious | 1.378.998 | .0521783 | 26.43 | 0.000 | 1.276.731 | 1.481.266 |
| Limitation legs not measured | -.4812845 | .4994244 | -0.96 | 0.335 | -1.460.138 | .4975694 |
| Limitation hands no | -.0400507 | .0304865 | -1.31 | 0.189 | -.0998031 | .0197016 |
| Limitation hands mild | .001923 | .0273927 | 0.07 | 0.944 | -.0517657 | .0556117 |
| Limitation hands moderate | .1094524 | .0300021 | 3.65 | 0.000 | .0506493 | .1682556 |
| Limitation hands serious | .1894756 | .0666454 | 2.84 | 0.004 | .058853 | .3200982 |
| Limitation hands not measured | -.2245576 | .5789708 | -0.39 | 0.698 | -135.932 | .9102043 |
| Limitation feet no | -.0869476 | .032226 | -2.70 | 0.007 | -.1501095 | -.0237857 |
| Limitation feet mild | .1354264 | .02559 | 5.29 | 0.000 | .085271 | .1855818 |
| Limitation feet moderate | .4448951 | .0274177 | 16.23 | 0.000 | .3911573 | .4986328 |
| Limitation feet serious | .5772386 | .0554633 | 10.41 | 0.000 | .4685326 | .6859446 |
| Limitation feet not measured | .9403446 | .5536938 | 1.70 | 0.089 | -.1448754 | 2.025.565 |
| time_id_1 | .4113576 | .0237885 | 17.29 | 0.000 | .364733 | .4579821 |
| time_id_2 | .3955317 | .0238228 | 16.60 | 0.000 | .3488399 | .4422235 |
| time_id_3 | .4032581 | .0233986 | 17.23 | 0.000 | .3573976 | .4491186 |
| time_id_4 | .4070752 | .023843 | 17.07 | 0.000 | .3603437 | .4538067 |
| time_id_5 | .4542091 | .0242144 | 18.76 | 0.000 | .4067498 | .5016683 |
| time_id_6 | .4136895 | .0236012 | 17.53 | 0.000 | .3674321 | .4599469 |
| time_id_7 | .4451939 | .0238914 | 18.63 | 0.000 | .3983677 | .4920201 |
| time_id_8 | .4894714 | .0245005 | 19.98 | 0.000 | .4414512 | .5374916 |
| time_id_9 | .429521 | .0241414 | 17.79 | 0.000 | .3822047 | .4768373 |
| time_id_10 | .4947819 | .0244715 | 20.22 | 0.000 | .4468186 | .5427452 |
| time_id_11 | .5250139 | .0241916 | 21.70 | 0.000 | .4775993 | .5724286 |
| time_id_12 | .6076847 | .0241865 | 25.12 | 0.000 | .5602801 | .6550894 |
| time_id_13 | .6661432 | .0240138 | 27.74 | 0.000 | .619077 | .7132094 |
| time_id_14 | .6822089 | .0242514 | 28.13 | 0.000 | .634677 | .7297409 |
| time_id_15 | .6884907 | .0237398 | 29.00 | 0.000 | .6419616 | .7350198 |
| time_id_16 | .7488455 | .0245277 | 30.53 | 0.000 | .700772 | .7969189 |
| time_id_17 | .7210639 | .0241372 | 29.87 | 0.000 | .6737557 | .768372 |
| time_id_18 | .7707145 | .0243838 | 31.61 | 0.000 | .7229232 | .8185058 |
| time_id_19 | .7806516 | .0244776 | 31.89 | 0.000 | .7326763 | .828627 |
| time_id_20 | .795515 | .024785 | 32.10 | 0.000 | .7469374 | .8440926 |
| time_id_21 | .7457283 | .0246724 | 30.23 | 0.000 | .6973713 | .7940853 |
| time_id_22 | .6861801 | .0244792 | 28.03 | 0.000 | .6382017 | .7341585 |
| time_id_23 | .7043325 | .0242384 | 29.06 | 0.000 | .656826 | .751839 |
| time_id_24 | .7419121 | .0243047 | 30.53 | 0.000 | .6942758 | .7895485 |
| time_id_25 | .701805 | .0238375 | 29.44 | 0.000 | .6550843 | .7485256 |
| time_id_26 | .7317337 | .024097 | 30.37 | 0.000 | .6845045 | .7789629 |
| time_id_27 | .7262499 | .0239711 | 30.30 | 0.000 | .6792673 | .7732325 |
| time_id_28 | .7543453 | .0245856 | 30.68 | 0.000 | .7061583 | .8025322 |
| time_id_29 | .7764502 | .0241133 | 32.20 | 0.000 | .7291889 | .8237115 |
| time_id_30 | .7818972 | .0242915 | 32.19 | 0.000 | .7342868 | .8295076 |
| time_id_31 | .7752309 | .0243209 | 31.88 | 0.000 | .7275628 | .8228991 |
| time_id_32 | .7656629 | .024644 | 31.07 | 0.000 | .7173615 | .8139644 |
| time_id_33 | .7366082 | .0249377 | 29.54 | 0.000 | .6877312 | .7854852 |
| time_id_34 | .7330047 | .0240531 | 30.47 | 0.000 | .6858615 | .7801478 |
| time_id_35 | .6859702 | .0241902 | 28.36 | 0.000 | .6385583 | .733382 |
| time_id_36 | .713759 | .0245589 | 29.06 | 0.000 | .6656245 | .7618936 |
| time_id_37 | .2824221 | .0237215 | 11.91 | 0.000 | .2359288 | .3289153 |
| time_id_38 | .2374472 | .0244211 | 9.72 | 0.000 | .1895827 | .2853117 |
| time_id_39 | .2527313 | .0242549 | 10.42 | 0.000 | .2051926 | .30027 |
| time_id_40 | .2595829 | .0241122 | 10.77 | 0.000 | .2123239 | .3068419 |
| time_id_41 | .2723892 | .0243116 | 11.20 | 0.000 | .2247393 | .3200392 |
| time_id_42 | .2682853 | .0245813 | 10.91 | 0.000 | .2201069 | .3164638 |
| time_id_43 | .3079552 | .0241297 | 12.76 | 0.000 | .2606619 | .3552485 |
| time_id_44 | .226741 | .0252554 | 8.98 | 0.000 | .1772413 | .2762406 |
| time_id_45 | .1864843 | .0251179 | 7.42 | 0.000 | .1372542 | .2357144 |
| time_id_46 | .1255067 | .0246054 | 5.10 | 0.000 | .0772811 | .1737324 |
| time_id_47 | .0811924 | .0257386 | 3.15 | 0.002 | .0307457 | .1316392 |
| Age2 | .0102846 | .0009937 | 10.35 | 0.000 | .008337 | .0122322 |
| Age | .0001319 | 7.15e-06 | 18.45 | 0.000 | .0001179 | .0001459 |
| Sex | .1733383 | .0484732 | 3.58 | 0.000 | .0783327 | .268344 |
| Personal care | -449.755 | .0099768 | -450.80 | 0.000 | -4.517.104 | -4.477.996 |
| Nursing home care | -418.262 | .0153727 | -272.08 | 0.000 | -421.275 | -4.152.491 |
| Care including a treatment | -3.368.188 | .0336611 | -100.06 | 0.000 | -3.434.163 | -3.302.214 |
| Care including stay | -.2524124 | .0735317 | -3.43 | 0.001 | -.396532 | -.1082928 |
| Counselling | -3.808.025 | .0156504 | -243.32 | 0.000 | -3.838.699 | -377.735 |
| Physical disability | 1.596.702 | .0777193 | 20.54 | 0.000 | 1.444.375 | 1.749.029 |
| Psychiatric/psychogeriatric | 1.397.389 | .0573186 | 24.38 | 0.000 | 1.285.047 | 1.509.732 |
| Somatic illness | 2.011.573 | .0598212 | 33.63 | 0.000 | 1.894.326 | 212.882 |
| Intellectual disability | 2.851 | .055698 | 51.19 | 0.000 | 2.741.834 | 2.960.166 |
| Reason not specified | -.4719109 | .0144836 | -32.58 | 0.000 | -.5002982 | -.4435235 |
| Reason new disease/limitation | -.25655 | .0143355 | -17.90 | 0.000 | -.2846471 | -.2284529 |
| Reason change disease/limitation | -.3061405 | .0148165 | -20.66 | 0.000 | -.3351802 | -.2771007 |
| Reason change living situation | .8185603 | .0226827 | 36.09 | 0.000 | .7741031 | .8630175 |
| Reason change informal care | .313444 | .0310934 | 10.08 | 0.000 | .2525021 | .3743859 |
| Reason end of validity LTC entitlement | -1.073.958 | .0141124 | -76.10 | 0.000 | -1.101.617 | -1.046.298 |
| Multi-person household | -1.639.413 | .0141008 | -116.26 | 0.000 | -166.705 | -1.611.776 |
| Household with partner/children | -2.490.819 | .0251475 | -99.05 | 0.000 | -2.540.107 | -2.441.531 |
| Independently + partner | -1.958.263 | .0111529 | -175.58 | 0.000 | -1.980.123 | -1.936.404 |
| Adult + 1 or more children | -2.287.111 | .0247508 | -92.41 | 0.000 | -2.335.622 | -2.238.601 |
| Alone and independently | -1.431.675 | .020431 | -70.07 | 0.000 | -1.471.719 | -1.391.631 |
| Child living with parents | -171.721 | .0099219 | -173.07 | 0.000 | -1.736.657 | -1.697.764 |
| Living situation was unknown | -1.749.648 | .0144328 | -121.23 | 0.000 | -1.777.936 | -1.721.361 |
| Age*Sex | -.0053422 | .000315 | -16.96 | 0.000 | -.0059596 | -.0047249 |
| Physical disability*Sex | .637611 | .0583019 | 10.94 | 0.000 | .5233414 | .7518806 |
| Psychiatric/psychogeriatric*Sex | .2810445 | .0469338 | 5.99 | 0.000 | .189056 | .373033 |
| Somatic illness*Sex | .2446719 | .0467887 | 5.23 | 0.000 | .1529677 | .336376 |
| Intellectual disability*Sex | -.0319828 | .0477663 | -0.67 | 0.503 | -.125603 | .0616374 |
| Physical disability*Age | .0007555 | .0010165 | 0.74 | 0.457 | -.0012368 | .0027479 |
| Psychiatric/psychogeriatric*Age | .0211527 | .0007488 | 28.25 | 0.000 | .0196851 | .0226203 |
| Somatic illness*Age | .0005977 | .0007751 | 0.77 | 0.441 | -.0009215 | .002117 |
| Intellectual disability*Age | .0044992 | .0008043 | 5.59 | 0.000 | .0029229 | .0060755 |
| _Cons | -.1564036 | .0606777 | -2.58 | 0.010 | -.2753297 | -.0374776 |
