# Supplementary material for: Practice variation in the Dutch long‐term care and the role of supply‐sensitive care: Is access to the Dutch long‐term care equitable?
Source: Health Econ. 2017 Mar 2;26(12):1728–42. doi: 10.1002/hec.3494 (PMC5811918; doi:10.1002/hec.3494)
Supplement: Supplementary file 1 — Data S1. Supporting info item [file HEC-26-1728-s001.zip › Graph 1 PV.doc]

**Graph 1. Percentages of IC entitlements granted relative to the number of inhabitants per care office region**
