# Supplementary material for: Practice variation in the Dutch long‐term care and the role of supply‐sensitive care: Is access to the Dutch long‐term care equitable?
Source: Health Econ. 2017 Mar 2;26(12):1728–42. doi: 10.1002/hec.3494 (PMC5811918; doi:10.1002/hec.3494)
Supplement: Supplementary file 1 — Data S1. Supporting info item [file HEC-26-1728-s001.zip › Graph 3 PV_.doc]

**Graph 3. Practice variation in the intensity of IC granted**

Observed < Predicted

Observed ~ Predicted

Observed > Predicted

The population average of € 150.10 is shown in the middle of the graph.
